# PIGO deficiency: palmoplantar keratoderma and novel mutations

**DOI:** 10.1186/s13023-017-0654-9

**Published:** 2017-05-25

**Authors:** Marie-Anne Morren, Jaak Jaeken, Gepke Visser, Isabelle Salles, Chris Van Geet, NIHR BioResource, Ilenia Simeoni, Ernest Turro, Kathleen Freson

**Affiliations:** 1 Departments of Dermatology, University of Leuven, Leuven, Belgium; 2 Pediatrics, University of Leuven, Leuven, Belgium; 3 Department of Pediatrics, Wilhelmina Children’s Hospital, Utrecht, The Netherlands; 4 Centre for Hematology, Imperial College, London, UK; 5 Department of Cardiovascular Sciences, Center for Molecular and Vascular Biology, University of Leuven, Leuven, Belgium; 6 NHS Blood and Transplant, Cambridge Biomedical Campus, Cambridge, UK; 7 Department of Haematology, University of Cambridge, Cambridge Biomedical Campus, Cambridge, UK; 8 Medical Research Council Biostatistics Unit, Cambridge Institute of Public Health, Cambridge Biomedical Campus, Cambridge, UK; 9 Department of Pediatrics, Center for Metabolic Diseases, University Hospital Gasthuisberg, Herestraat 49, BE 3000 Leuven, Belgium; 10 NIHR BioResource - Rare Diseases, Cambridge University Hospitals, Cambridge Biomedical Campus, Cambridge, UK

**Keywords:** CDG, Congenital disorder(s) of glycosylation, Glycosylphosphatidylinositol, GPI, Hyperkeratosis, Hyperphosphatasemia, PIGO-CDG, Platelet dysfunction

## Abstract

**Background:**

Several genetic defects have been identified in the glycosylphosphatidylinositol (GPI) anchor synthesis, including mutations in *PIGO* encoding phosphatidylinositol glycan anchor biosynthesis class O protein. These defects constitute a subgroup of the congenital disorders of glycosylation (CDG). Seven patients from five families have been reported carrying variants in *PIGO* that cause an autosomal recessive syndrome characterised by dysmorphism, psychomotor disability, epilepsy and hyperphosphatasemia.

**Methods:**

Whole exome sequencing was performed in a boy with dysmorphism, psychomotor disability, epilepsy, palmoplantar keratoderma, hyperphosphatasemia and platelet dysfunction without a clinical bleeding phenotype.

**Results:**

Two novel variants in *PIGO* were detected. The missense variant encoding p. His871Pro was inherited from the boy’s father while the frameshift variant encoding p. Arg604ProfsTer40 was maternally inherited.

**Conclusion:**

A boy with two novel *PIGO* variants is reported. The skin phenotype and platelet dysfunction in this patient have not been described in previously reported patients with PIGO deficiency but it is of course uncertain whether these are caused by this disorder. The literature on PIGO deficiency is reviewed.

**Electronic supplementary material:**

The online version of this article (doi:10.1186/s13023-017-0654-9) contains supplementary material, which is available to authorized users.

## Introduction

GPI anchors are a group of glycolipids with a glycan core, a phosphoethanolamine linker and a phospholipid tail. At least 150 human cell-surface proteins are posttranslationally modified by GPI anchors at the carboxyl-terminus. These proteins are anchored to the outer leaflet of the plasma membrane via the phosphatidylinositol moiety. These GPI-anchored proteins include adhesion molecules, complement regulatory proteins, hydrolytic enzymes, protease inhibitors and receptors. At least 26 genes are involved in the biosynthesis, protein-attachment and remodelling of mammalian GPI [1]. Genetic defects have been reported in 12 of these genes [2, 3]. They belong to subgroup 3 in the current classification of congenital disorders of glycosylation (CDG) [4]. One of them is a defect in phosphatidylinositol glycan anchor biosynthesis class O protein (PIGO). This enzyme, together with PIGF, catalyzes the attachment of ethanolamine phosphate to the third mannose of the three-mannosyl glycan core of GPI. Seven patients have been reported from five families with PIGO deficiency [5–8]. The present report is on a patient who expands the phenotypic spectrum of PIGO deficiency and carries novel mutations in the PIGO gene.

## Patient report

The boy was born in 1994 from unrelated healthy parents from Afro-Caribbean ancestry after a 34 weeks pregnancy. He has a healthy sister. The family history was negative for keratotic disorders. Pregnancy was complicated by polyhydramnion from the fourth month. Delivery was normal. Birth weight was 2870 g, length unknown and head circumference 36.5 cm. At birth, he showed oedema, especially of the distal ends of the extremities, as well as dysmorphism: broad nasal bridge, right preauricular tag, W-shaped upper lip, relatively large mouth, fusion of upper medial incisors, short neck, hypogenitalism and hypogonadism (testicles not palpable), and hypoplastic toenails. At 6 weeks, a deep infantile hemangioma was noted on the right upper thorax. At 4 months, he was operated for a right inguinal hernia. He had focal epilepsy at the age of 17 months, which changed to multifocal epilepsy and was reasonably controlled with valproate. Both motor and cognitive development were severely disabled. He started to walk at 3.5 years (abnormal gait with abduction of both feet), and said ‘mama’ and ‘papa’ at about 6 years.

On physical examination at 7 years, height was 123 cm (SDS 0.0), weight 25.5 kg (SDS 0.6), head circumference 56 cm (SDS 2.5). He smiled all the time. Moderate obesity, convergent strabism and general hyperlaxity were noted but no hypotonia. There was a residual hemangioma on the right upper thorax. Dysmorphism included malalignment of the upper medial incisors (no fusion of the permanent teeth), a broad nasal bridge, coarse ears, widely spaced nipples, mild camptodactyly of the fingers, and small hands and feet with short fingers and toes. There was a striking hyperkeratosis of the foot soles and hand palms.

**Fig. 1:**
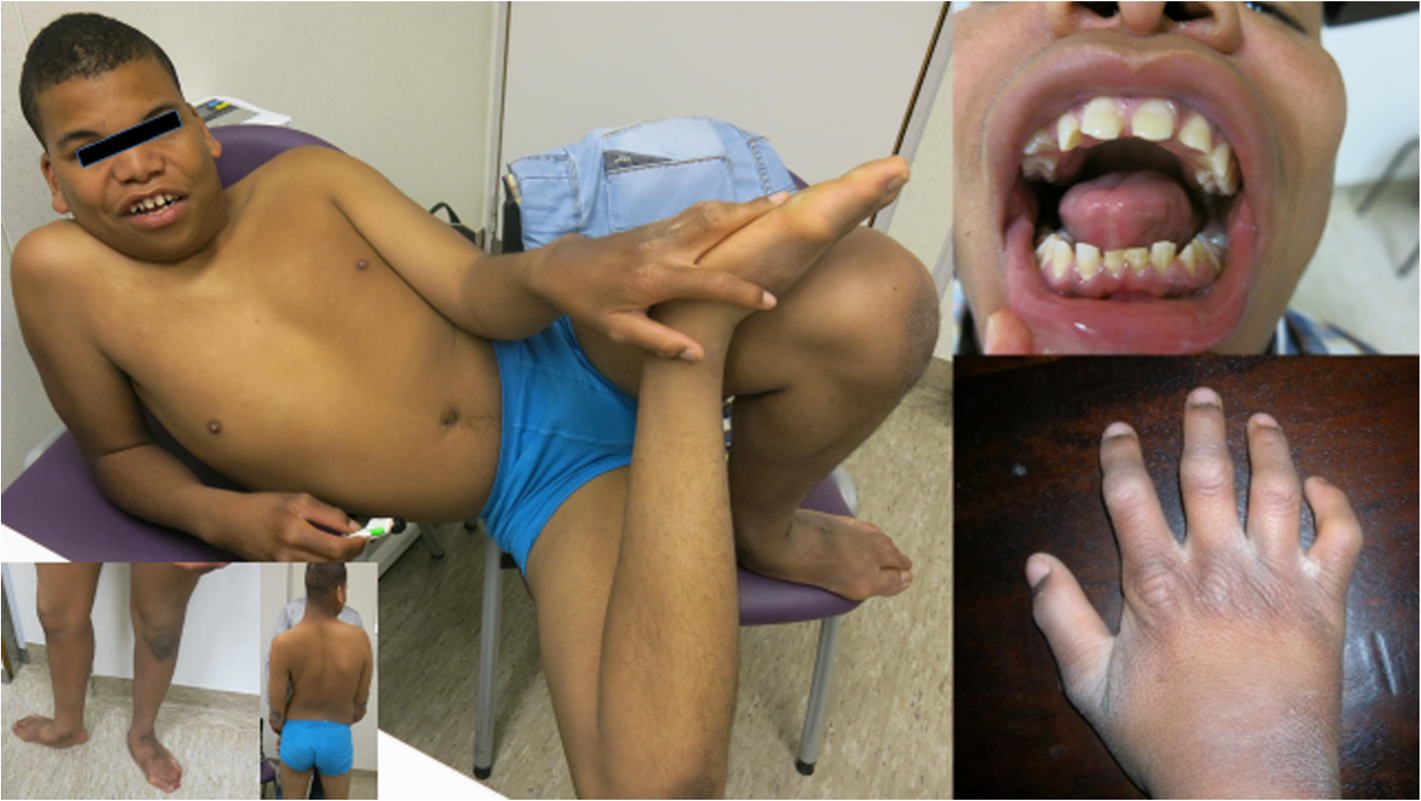
The patient at age 13 years. Note facial dysmorphism, exorotation of the feet and keratoderma of the dorsum of the *right hand*

**Fig. 2:**
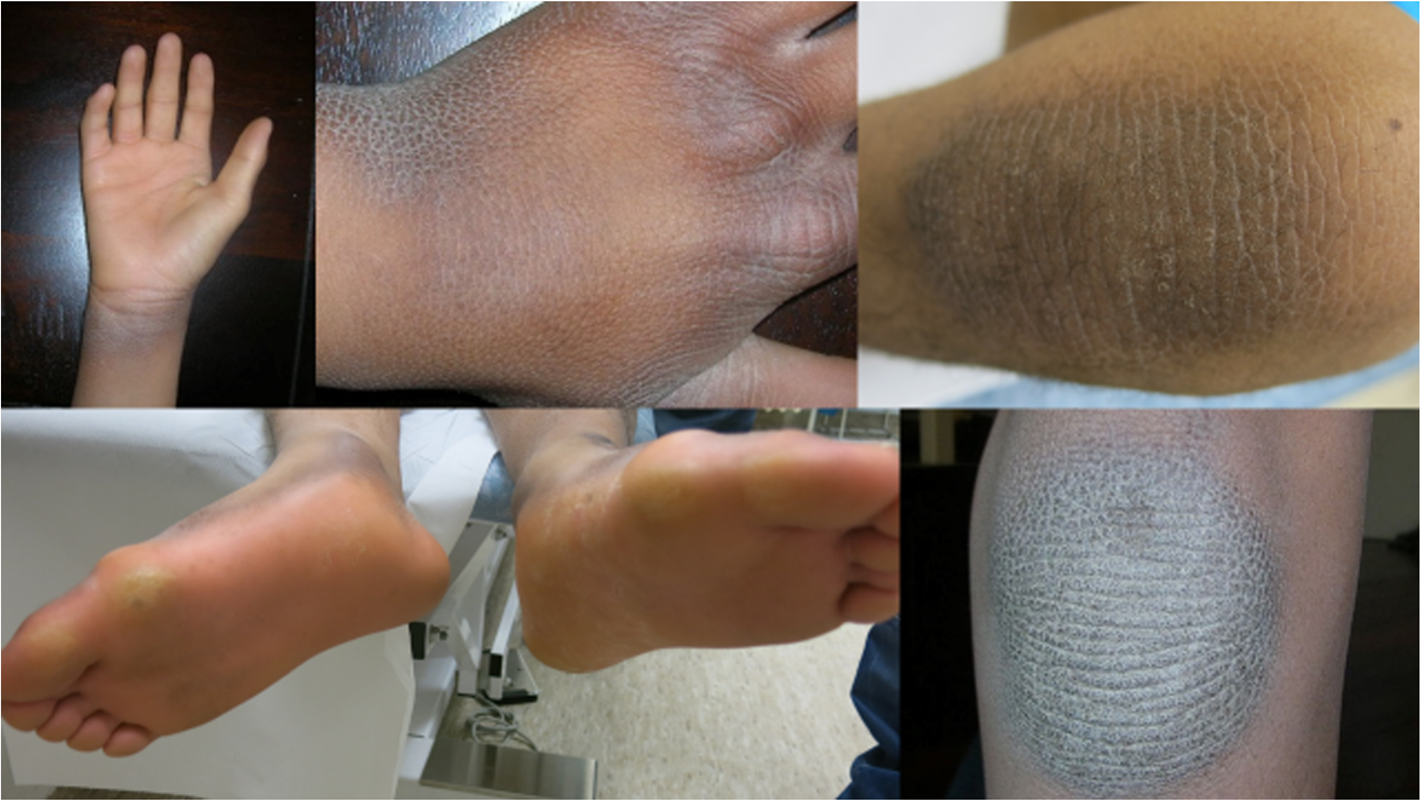
Keratoderma of the hands, foot soles and knees of the patient at 13 years

Physical examination at 11.5 years showed a height of about 144 cm (SDS – 0.7), weight 40 kg (SDS + 0.2) and head circumference 57 cm (SDS + 1.9). He was still incontinent for urine and faeces. The father mentioned progress in language understanding but speech was absent and he made himself understandable with gestures. He walked with his feet in 90 degrees abduction. There was a thickened palmar and plantar skin, more pronounced on pressure points, with a fine scaling extending over the dorsal side of hands and feet including the wrists and ankles. This thickened skin showed accentuation of the skin lines, particularly on the wrists. On the extensor side of elbows and knees the same thickened skin was seen (Figs. 1 and 2). Vision and hearing were clinically normal. There was no hepatosplenomegaly. Cardiopulmonary examination was normal. The testes were small (walnut size). Tendon reflexes were brisk. He had a mild anal prolapse on pressure.

Laboratory investigation showed persistently increased serum alkaline phosphatase (last control at 13 years: 5131 U/L; normal < 720) mainly due to increased bone isozyme. There was also a decreased serum apolipoprotein B (0.42 g/L; normal range 0.66–1.33) and LDL-cholesterol (37 mg/dL) with normal HDL cholesterol and triglycerides as well as a mild increase of serum amylase (121 U/L; normal range 28–100) and thyroid stimulating hormone (6.64 mIU/L; normal range 0.27–4.20) but with normal free T4 and thyroxin-binding globulin levels. Further routine blood and urine chemistry as well as metabolic screening were normal including serum calcium, phosphate, transaminases, creatine kinase, lactate dehydrogenase, factor XI, ceruloplasmin, cholinesterase, lipase, IgA, IgG, IgM and serum transferrin isoelectrofocusing.

**Fig. 3:**
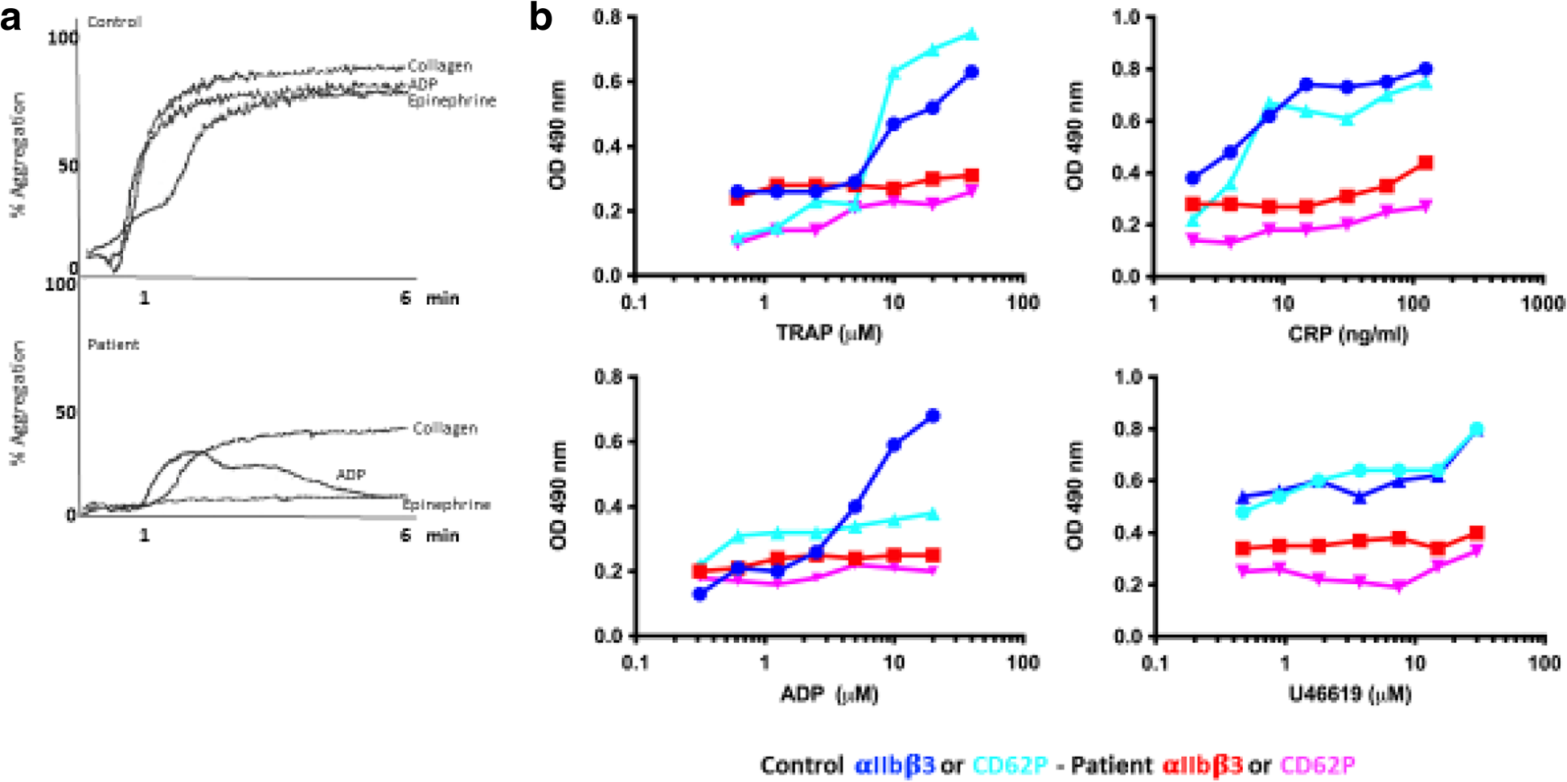
Platelet function studies. **a** Platelet aggregation with Horm collagen (2 μg/ml), ADP (5 μM) and epinephrine (5 μg/ml) was reduced in the patient. **b** A high throughput ELISA was performed that records dose response activation of platelets with monoclonal antibodies against P-selectin (CD62P) or αIIbβ3 to detect platelet activation with ADP, U46619, TRAP and CRP at different concentrations

The patient had no obvious clinical bleeding problem and his Ivy bleeding time was normal as were all blood cell counts. Repeated platelet function testing showed decreased aggregation responses for ristocetin, ADP, epinephrine and Horm collagen (Fig. 3a). A similar multi - agonist platelet activation defect was detected using a high-throughput ELISA that records dose response activation of platelets where monoclonal antibodies against P-selectin (CD62P) or αIIbβ3 and GPIbα were used to capture and detect platelet activation with ADP, U46619, TRAP and CRP [9] (Fig. 3b). In contrast, ATP secretion from dense granules after stimulation with 2 μg/ml Horm collagen was normal (4.1 and 4.8 μM; normal range of 2–7 μM). As mentioned above, the patient is on valproate therapy that is known to have slight effects on the arachidonic pathway of platelets. However, the observed broad platelet activation defect of this patient is not compatible with a defect in the arachidonic pathway. It was not possible to withdraw the drug for further platelet testing.

Radiological examination of the skeleton showed a thin cortex, triangular distal toe phalanges, scoliosis, wedge-shaped anterior flattening of the vertebrae, and a fusion anomaly of the arcus posterior of D3. MRI of the brain at 6 years showed enlarged lateral ventricles, a cavum septum pellucidum, a thin corpus callosum, and minimal white matter lesions at the level of the posterior horns.

A skin biopsy at the elbow showed a “basket woven” hyperorthokeratosis with papillomatosis. Electronmicroscopy revealed clear vesicles but this might have been a technical artefact.

DNA from the patient was analysed by whole exome sequencing (WES) as part of the NIHR BRIDGE-Bleeding and Platelet Disorders study [10] and compound heterozygous (and X-linked) rare variants were found in 8 genes that included two previously unreported variants in the *PIGO* gene for phosphatidylinositol glycan anchor biosynthesis class O protein. Details on gene selection from the WES data is described in the supplementary data (Additional file 1). Interestingly, recessive *PIGO* variants cause hyperphosphatasemia with mental retardation syndrome 2 (MIM 614749), as found in the patient. The missense variant c.2612A > C (p.His871Pro) was absent from ExAC while the frameshift variant c.1810dupC (p.Arg604ProfsTer40) was present in ExAC at a minor allele frequency (MAF) of 0.0001 (0.0002 in non-Finnish Europeans). Sanger sequencing confirmed the variants in the child and the mother was the carrier of the variant encoding p.Arg604ProfsTer40 while DNA from the father showed he carried the variant encoding p.His871Pro. Different DNA variant scoring systems predict p.His871Pro as pathogenic with CADD score of 20, PolyPhen score of 0.836 (‘possibly damaging’), SIFT score of 5.44 (‘deleterious’) and GERP score of 5.44.

## Discussion

To date, only 7 patients from 5 families have been described with PIGO deficiency (Table 1) [5–8]. Interestingly, they all carry the combination of a high impact variant with a missense variant in *PIGO*. Table 1 summarizes the clinical findings of the previously reported patients and our patient. All eight patients had a moderate or severe psychomotor disability as well as hyperphosphatasemia. The following symptoms were present in the majority of patients: epilepsy (6/8), facial dysmorphism (6/8), brachytelephalangy (5/8), nail hypoplasia (4/7), and anorectal abnormalities (5/6). A minority of patients showed various neurological, cardiac, urogenital and skeletal abnormalities. Novel features observed in our patient include palmoplantar keratoderma, extreme rotation of the feet, dental abnormalities and platelet dysfunction.

**Table 1:** Summary of clinical features and mutations in PIGO-deficient patients

	Krawitz et al. (pt A1)	Krawitz et al. (pt A2)	Krawitz et al. (pt B1)	Kuki et al.	Nakamura et al. (pt 1)	Nakamura et al. (pt 2)	Xue et al.	Present report
Age	15 years at publication	12 years at publication	Died at 22 months	9 years at publication	19 years at publication	Died at 1 year	26 months at publication	22 years at present
Gender	Female	Female	Female	Male	Male	Female	Male	Male
Psychomotor disability	Severe	Moderate	Severe	Moderate	Severe	Severe	Moderate/severe	Moderate
Microcephaly	NA	NA	+	+	+	NA	Not at birth	-
Epilepsy	-	-	+	+	+	+	+	+
Facial dysmorphism	Hypertelorism, wide-set eyes, long palpebral fissures, short nose, broad nasal bridge and tip, tented mouth	Hypertelorism, wide-set eyes, long palpebral fissures, short nose, broad nasal bridge and tip, tented mouth	Facial asymmetry, hypertelorism, wide-set eyes, long palpebral fissures, short nose, broad nasal bridge and tip, tented mouth, large ears with fleshy and uplifted earlobes	Coarse face, hypertelorism, blepharophimosis, short nose, broad nasal bridge, L cleft lip, low-set ears	High arched palate, tented mouth	-	-	Broad nasal bridge, relatively large mouth, fusion of upper medial milk incisors, W-shaped upper lip, coarse ears, R preauricular tag
Brachytelephalangy	+	+	+	+	-	-	-	+
Nail hypoplasia	Fingers and toes	Fingers and toes	Fingers and toes	NA	-	-	-	Toes
Anorectal abnormalities	Anal stenosis	Anal stenosis	Anal atresia with perineal fistula	Hirschsprung disease	NA	NA	-	Mild anal prolapse on pressure
Hyperphosphatasemia	+	+	+	+	+	+	+	+
Other features	Growth retardation, broad halluces, vesicoureteral reflux	Broad halluces, atonic bladder	Growth retardation, L coronal synostosis, broad halluces, atrial septal defect, peripheral pulmonary synostosis, enlarged supratentorial ventricular system	Deafness, hypotonia, tetralogy of Fallot, brain hypomyelination	Chorea, hypotonia, abnormal auditory brain response, cerebral and cerebellar atrophy at 6 years		Brain diffusion-weighted magnetic resonance imaging: slightly high signal in globus pallidus and dorsal brain stem	Macrocephaly, convergent strabism, palmoplantar keratoderma, hemangioma R upper thorax, exorotation of the feet, hypotonia, hyperlaxity, camptodactyly of fingers, hypogonadism, enlarged lateral ventricles, thin corpus callosum, small white matter lesions
Mutations: cDNA/protein	c.2869C > T/p.Leu957Phe c.2361dupC/p.Thr788Hisfs*5	c.2869C > T/p.Leu957Phe c.2361dupC/p. Thr788Hisfs*5	c.2869C > T/p.Leu957Phe c.3069 + 5G > A	c.355C > T/p.Arg119Trp c.23497_23498del/p.Ala834fs	c.389C > A/p.Thr130Asn c.1288C > T/p.Gln430*	c.389C > A/p.Thr130Asn c.1288C > T/p.Gln430*	c.458 T > C/p.Phe153Ser c.1355_1356del/p.Ala452Glyfs*52	c.2612A > C/p.His871Pro c.1810dupC/p.Arg604ProfsTer40

*NA* not available

Cutaneous abnormalities have been reported in two other GPI anchor synthesis disorders. In patients with PIGA deficiency, dry scaling, ichthyosis-like and eczema-like lesions, pigmentation abnormalities, and linear plaque-like scales, including the feet, have been described [11]. More consistent skin lesions have been reported in patients with PIGL deficiency or CHIME syndrome (coloboma, congenital heart defects, early onset migratory ichthyosiform dermatosis, mental retardation, and ear anomalies, including conductive hearing loss). A diffuse, erythematous, pruritic, often migratory rash at or shortly after birth (sometimes even erythroderma) was present in all described cases. Thereafter, the skin becomes increasingly ichthyotic; primarily at the flexural surfaces [12, 13]. GPI anchoring plays a role in skin cells, particularly in keratinocytes. An epidermal-specific defect of GPI anchor in Pig-a null mice results in Harlequin ichthyosis-like features [14, 15]. In these mice, there was an impaired processing of profilaggrin to filaggrin, accompanied by a decreased activity of protein phosphatase 2A involved in this processing. Proteasin, one of the enzymes involved in filaggrin synthesis, is a GPI-anchored protein, and in mice its deficiency leads to a phenotype comparable to matriptase deficiency, a cause of autosomal recessive ichthyosis with hypotrichosis [15]. On the other hand, Tam et al. have shown that GPI-anchored proteins regulate transforming growth factor-beta signalling in human keratinocytes [16].

Platelet function has not been studied in GPI anchor disorders, probably because until now no patient showed a haematological phenotype. We studied it in our patient as part of the etiological work-up in unexplained psychomotor disability. A characteristic feature of PIGO deficiency (and of six other known GPI anchor synthesis defects, namely in PIGA, PIGV, PIGW, PGAP1, PGAP2 and PGAP3) is the increase of serum alkaline phosphatase (tissue nonspecific; liver/bone/kidney), while in PIGT deficiency serum alkaline phosphatase is decreased [17] (reminiscent of hereditary hypophosphatasia). Alkaline phosphatase testing may be helpful in the etiological work-up of patients with (syndromic or non-syndromic) intellectual disability, and the finding of increased or decreased levels of this enzyme should prompt a search for a GPI anchor defect.

Coagulation and platelets defects have been reported in different congenital disorders of glycosylation as important receptors and proteins for coagulation, platelet formation and function are regulated by glycosylation [18–25]. The importance of GPI-anchoring for platelet proteins is not well studied. It is known that GPI-anchored glycoproteins are absent or deficient in platelets from patients with paroxysmal nocturnal haemoglobinuria (PNH) [26]. PNH is an acquired stem cell disorder due to somatic variants in *PIGA* and causes an abnormal susceptibility of erythrocytes to complement induced lysis, resulting in episodes of intravascular haemolysis, haemoglobinuria and both thromboembolic events and bleeding complications. Platelets from PNH cases showed platelet hyporeactivity using in vitro assays possibly due to chronic hyperstimulation in the circulation [27]. Though functional platelet defects have not been reported in the other patients with *PIGO* variants, we detected a multi- agonist platelet defect in our patient as a subclinical phenotype. Further studies need to be undertaken to support these findings and compare them with PNH.

## Additional file

Additional file 1: Gene selection from WES data. (DOCX 105 kb)

## Abbreviations

CDG: Congenital disorder(s) of glycosylation
GPI: Glycosylphosphatidylinositol
PIGO: Phosphatidylinositol glycan anchor biosynthesis class O protein
PNH: Paroxysmal nocturnal hemoglobinuria
