# Supplementary material for: PIGO deficiency: palmoplantar keratoderma and novel mutations
Source: Orphanet J Rare Dis. 2017 May 25;12:101. doi: 10.1186/s13023-017-0654-9 (PMC5445308; doi:10.1186/s13023-017-0654-9)
Supplement: Additional file 1: — Gene selection from WES data. (DOCX 105 kb) [file 13023_2017_654_MOESM1_ESM.docx]

**Gene selection from WES data**

As parents were asymptomatic, we analysed the WES for autosomal recessive (AR) variants. We cannot exclude the role of pathogenic ‘*de* novo’ variants in our patient as DNA from the parents was not analysed by WES.

Variant filtering was done to include LoF and missense variants are absent from ExAC or are present with frequency of less than 0.001 and also have a CADD score (1) of at least 15. This resulted in 7 compound heterozygous and 1 hemizygous defect. Selection was further made using pathogenicity scores and the analysis of other AR patients from NIHR-BioResource Rare Diseases (RD) project (total of 7600) for these 8 candidate genes. The PIGO variants could explain the phenotype of the patients and the combination of having a LOF variants with a missense seems to be present in all known PIGO patients (Table1).

**Table with 7 compound heterozygous and 1 hemizygous variants present in the patient.**

| **Gene name** | **MAF in ExAC** | **Variant** | **Effect at protein level** | **CADD** | **PolyPhen** | **SIFT** | **Further interpretation** |
| --- | --- | --- | --- | --- | --- | --- | --- |
| CYP4A22 | 0.0008813  (1 homozygous) | 1:47603256 G/C (rs141897799) | Lys33Asn | 17.81 | Benign | Deleterious | 3 RD patients carry the same 2 variants but have other phenotypes (in cis ?). |
|  | 0.0009719  (1 homozygous) | 1:47607827 C/T (rs147010308) | Arg144Trp | 24.4 | Benign | Deleterious |  |
| ALMS1 |  |  | Glu17Lys | 24.6 |  |  | False positive variants |
|  |  |  | Glu19Lys | 23.9 |  |  |  |
| BMP3 | 0.00002  coverage problem for this variant | 4:81952440 T/C | Met1? | 20 |  |  | No other AR patient. |
|  | 0.0009556 | 4:81967618 C/T (rs145297209) | Thr348Met | 24 | Possible damaging | Tolerated |  |
| LETM2 | 0.0001223 | 8:38251751 T/C | Ser213Pro | 27 | Possibly damaging | Deleterious | 2 RD patients carry the same 2 variants but have other phenotypes (in cis?). These variants are only present in the transcript ENST00000379957 of this gene while not in the major transcript. |
|  | 0.0002883  (1 homozygous) | 8:38257914 C/G (rs147828005) | Ser257Cys | 23 | Possibly damaging | Deleterious |  |
| PIGO | absent | 9: 35091272 T/G | His871Pro | 25 | Possibly damaging | Deleterious | 2 RD patients with different phenotypes have AR missense variants but not in combination with LOF variant. These AR variants can also be in cis. |
|  | 0.0001318 | 9:35092073 C/CG (rs144507866) | Arg604ProfsTer40 | 28.2 |  |  |  |
| SPTBN5 | 1,65E-02 | 15:42160706 T/G | Ser1983Arg | 17.97 | Benign | ? | 33 RD patients have AR variants but other phenotypes |
|  | 0.0003145 | 15:42185117 C/T | Arg120Gln | 24 | probably_damaging | ? |  |
| PMFBP1 | 0.000313 | 16:72170400 C/G (rs149950926) | Glu384Gln | 22.4 | probably_damaging | Tolerated | 2 RD patients with AR variatns and different phenotypes |
|  | 0.0001071 | 16:72174366 T/C (rs148861582) | Gln251Arg | 23.1 | Benign | Deleterious |  |
| RAB40AL  (Xlinked) | 0.0001483  (1 hemizygous) | X:102192946 A/G (rs146508617) | Arg234Gly | 23.5 | probably_damaging | Tolerated | 3 RD patients with hemizygous variants and different phenotypes |

RD: Rare disease

1. Kircher M, Witten DM, Jain P, O'Roak BJ, Cooper GM, Shendure J. A general framework for estimating the relative pathogenicity of human genetic variants. Nat Genet. 2014;46:310-5.
